# Concentration-dependent dual effects of exogenous sucrose on nitrogen metabolism in *Andrographis paniculata*

**DOI:** 10.1038/s41598-022-08971-x

**Published:** 2022-03-22

**Authors:** Xue-Jing Huang, Shao-Fen Jian, Dong-Liang Chen, Chu Zhong, Jian-Hua Miao

**Affiliations:** 1 Pharmaceutical College, Guangxi Medical University, Nanning, 530021 China; 2 Guangxi Key Laboratory of Medicinal Resources Protection and Genetic Improvement, Guangxi Botanical Garden of Medicinal Plants, Nanning, 530023 China; 3 Guangxi Engineering Research Centre of TCM Intelligent Creation, Guangxi Botanical Garden of Medicinal Plants, Nanning, 530023 China

**Keywords:** Plant physiology, Plant signalling

## Abstract

The effects of exogenous sucrose (Suc) concentrations (0, 0.5, 1, 5, 10 mmol L^−1^) on carbon (C) and nitrogen (N) metabolisms were investigated in a medicinal plant *Andrographis paniculata* (Chuanxinlian). Suc application with the concentration of 0.5–5 mmol L^−1^ significantly promoted plant growth. In contrast, 10 mmol L^−1^ Suc retarded plant growth and increased contents of anthocyanin and MDA and activity of SOD in comparison to 0.5–5 mmol L^−1^ Suc. Suc application increased contents of leaf soluble sugar, reducing sugar and trerhalose, as well as isocitrate dehydrogenase (ICDH) activity, increasing supply of C-skeleton for N assimilation. However, total leaf N was peaked at 1 mmol L^−1^ Suc, which was consistent with root activity, suggesting that exogenous Suc enhanced root N uptake. At 10 mmol L^−1^ Suc, total leaf N and activities of glutamine synthase (GS), glutamate synthase (GOGAT), NADH-dependent glutamate dehydrogenase (NADH-GDH) and glutamic–pyruvic transaminase (GPT) were strongly reduced but NH_4_^+^ concentration was significantly increased. The results revealed that exogenous Suc is an effective stimulant for *A. paniculata* plant growth. Low Suc concentration (e.g. 1 mmol L^−1^) increased supply of C-skeleton and promoted N uptake and assimilation in *A. paniculata* plant, whereas high Suc concentration (e.g. 10 mmol L^−1^) uncoupled C and N metabolisms, reduced N metabolism and induced plant senescence.

## Introduction

Sucrose (Suc) is one of the primary free sugar products of plant photosynthesis and the main form of sugar transporting between plant tissues and cells. It provides substrates and energy for plant growth, development and biosynthesis of important compounds such as protein, cellulose, and starch. It is also a critical signal compound regulating plant growth and development and the expression of genes cross-talk with phytohormones and defensive signals^1–4^. Suc is closely related to plant nutrition responses. High Suc accumulation in plant tissues induces plant nitrogen (N)-, phosphorus (P)-, and iron (Fe)-deficiency responses^3,5,6^, in which process it has been identified as a vital signal^7,8^.

Carbon (C) and N metabolisms, the two most important and intimately connected fundamental metabolic processes in plants, play important roles in plant growth and development and adaptation to adversities^9,10^. Exogenous application of sugars to change the C and N status of plants provides an approach for studying the effects of C and N metabolism on plant development. Exogenous application of Suc has been suggesting as an effective strategy for improving crop quality^11^ and mitigating detrimental effects of environmental stresses such as drought^12^, salt stress^13,14^, and extreme temperature^14,15^. The attenuating effect of exogenous Suc on plants exposed to stress conditions was associated with increased antioxidant capacity^13^, osmotic potential balance^16^ and amelioration of C and N metabolisms^17^.

In normal growth conditions, the effects of exogenous Suc on plant growth and metabolism vary greatly depending upon plant species, duration of treatment, and growth conditions. Suc improved the levels of carbohydrate and nitrogenous compounds in plants by increasing the activities of C and N metabolic enzymes^11,18^. However, in some species such as radish and sugarcane, sugar metabolites, photosynthesis, and nitrogenous compounds were down-regulated by exogenous application of Suc^19–21^. Source-sink equilibrium affects the response of plants to exogenous Suc^22^. Exogenous Suc displayed promoting effects when source Suc is a major limiting factor, while in sink limitation conditions exogenous Suc induced strong accumulation of hexose and starch, followed by inhibition of photosynthesis^22^.

*Andrographis paniculata* (known as ‘Chuanxinlian’ in China) is an important traditional medicinal plant which is widely used in China, India, and Southeast Asian countries for therapeutic values^23^. Its main bioactive ingredient andrographolide has great medical values in anti-inflammatory, antibacterial and antiviral^24^. The leaf of this plant is the major organ of andrographolide biosynthesis. Therefore, to understand the interaction of C and N metabolism in plants is of great importance to regulate plant growth and improve the productivity of Chuanxinlian herbs. In the current study, Chuanxinlian plants were treated with different Suc concentrations nutritionally, and the C and N metabolisms in leaves were determined. We aimed to establish the foundation to reveal the role of Suc in modulation of Chuanxinlian plant growth.

## Materials and methods

### Materials and treatment

An Acanthaceae annual medicinal plant *Andrographis paniculata* was used in this study. The plant study complies with relevant institutional, national, and international guidelines and legislation. The seeds were provided by the seed bank of the Guangxi Botanical Garden of Medicinal Plants. The seeds were germinated on wet filter paper at room temperature (about 30 °C) and then transferred to a pearlite–vermiculite matrix for continuous growth. When the seedlings were two-leaf age, they were transplanted to pots grown in perlite–vermiculite matrix and fed with nutrient solution till four-leaf age. Then the seedlings were separated into five groups and treated with 0 (control), 0.5, 1, 5, and 10 mmol L^−1^ exogenous Suc, respectively, by adding this chemical in nutrient solution, for a month.

The nutrient solution contained 3 mmol L^−1^ urea (6 mmol L^−1^ N), 0.4 mmol L^−1^ NaH_2_PO_4_, 2 mmol L^−1^ KCl, 0.5 mmol L^−1^ MgSO_4_, 2 mmol L^−1^ CaCl_2_, 18 μmol L^−1^ H_3_BO_3_, 0.1 μmol L^−1^ (NH_4_)_6_Mo_7_O_24_, 0.15 μmol L^−1^ CuSO_4_, 0.15 μmol L^−1^ ZnSO_4_, 3.5 μmol L^−1^ MnSO_4_, and 1.25 μmol L^−1^ Fe-EDTA. The pH of the nutrient solution was adjusted to 6.0, and 10 mg L^−1^ benzylpenicillin was added to prohibit reproduction of microbes. The seedlings were supplied with nutrient solution twice a week and 200 mL per pot.

The experiment was arranged in a completely randomized design with five treatments, and each treatment had eight independent pots, each of which consisted of two plants.

### Root activity measurement

Fresh fibrous roots were cut off 2 cm from the root tip, and root activity was measured by a triphenyl tetrazolium chloride (TTC) method^25^. Briefly, the fresh cut living root samples were immersed immediately in a 0.2% TTC solution in 0.1 mol L^−1^ phosphate buffer (pH 7.0) for 3 h at 30 °C in the dark. The generated trimethoprim in the roots was extracted with ethyl acetate by thoroughly grinding the samples. The extract was measured spectrophotometrically at 485 nm by a spectrophotometer (L5, Shanghai Yifen Scientific Instrument Co., Ltd, China; the same as below), and root activity was presented as the generation rate of tetrazole reductive compounds per fresh root weight (mg g^−1^ h^−1^).

### Chlorophyll and anthocyanin measurements

Fresh leaf samples (100 mg) were immersed in 25 mL mixture of alcohol and acetone (v:v = 1:1) at room temperature in the dark for 24 h till the leaves were completely white. The concentrations of chlorophyll *a* and *b* in the extracts were calculated from the absorbance at 663 nm (A_663_) and 645 nm (A_645_)^26^:

1 $$ {\text{C}}a = {12}.{7} \times {\text{A}}_{{{663}}} - {2}.{69} \times {\text{A}}_{{{645}}} $$

2 $$ {\text{C}}b = {22}.{9} \times {\text{A}}_{{{645}}} - {4}.{68} \times {\text{A}}_{{{663}}} $$

where *Ca* and *Cb* were the concentration of chlorphyll *a* and *b*, respectively.

Anthocyanin in fresh leaves was extracted by immersing the samples in acid ethanol solution (0.1 mol L^−1^ HCl in 95% ethanol) at 60 °C for 1 h. The absorbance of the extract was measured at 530 nm, 620 nm, and 650 nm, respectively. Anthocyanin content based on fresh weight was calculated using the molar extinction coefficient of 4.62  × 10^4^^27^.

### Malondialdehyde (MDA) content and superoxide dismutase (SOD) activity

Fresh leaf samples were frozen in liquid nitrogen, powdered with liquid nitrogen, and stored at − 80 °C for physiological and biochemical assays.

Frozen leaf samples (100 mg) were homogenized in precooled mortar and pestle with 4 mL 0.1 mol L^−1^ sodium phosphate buffer (pH 7.8). The homogenate was centrifuged at 4 °C and 10,000×*g* for 10 min by an Eppendorf centrifuge (5424R, Eppendorf, Germany; the same as below), and the supernatant was used for MDA and SOD assays. MDA was measured colorimetrically at 600 nm, 532 nm, and 450 nm, respectively, as in Hodges et al.^28^, and the MDA concentration was calculated using the molar extinction coefficient of 0.155 mM^−1^ cm^−1^. MDA content was represented by the amount of thiobarbituric acid reactive substances (TBARS) per fresh root weight (nmol g^−1^ Fw).

SOD activity was determined according to the method described in Giannopolitis and Ries^29^. After adding 0.1 mL enzyme extraction in the reaction system containing 25 mmol L^−1^ sodium phosphate buffer (pH 7.8), 13 mmol L^−1^ methionine, 2 mmol L^−1^ riboflavin, 10 mmol L^−1^ EDTA-Na_2_, and 75 mmol L^−1^ nitro blue tetrazolium (NBT), the mixtures were put under light conditions (PAR = 200 μmol m^−2^ s^−1^) for 20 min and then measured chromometrically at 560 nm.

### Sugars and enzyme activities

Sugars in frozen leaf samples (100 mg) were extracted using deionized water in 80 °C water bath for 30 min, and followed by centrifuged at 10,000×*g* for 10 min after cooling. The sucrose in the supernatant was determined by the resorcinol-spectrophotometric method as described in Li et al.^30^. Sucrose was used as standard. Total soluble sugar was measured colorimetrically by sulphate-anthrone method at 620 nm^31^. Reducing sugar was measured using the method of 3,5-dinitrosalicylic acid (DNS) by colorimetric assay at 540 nm^32^. Glucose was used as standard for total soluble sugar and reducing sugar measurements.

Trehalose in leaves was measured by the anthrone-sulfuric acid method using trehalose as standard^33^. About 100 mg frozen leaf samples were extracted with deionized water at 4 °C by grinding. After centrifuging at 10,000×*g* and 4 °C for 10 min, trehalose in the supernatant was reacted with 0.2% anthrone in 85% sulfuric acid solution (the volume ratio of supernatant:anthrone solution was 1:3) in boiling water bath for 10 min, and then quantified colorimetrically at 625 nm.

Invertase was extracted by homogenizing frozen leaf samples with precooled deionized water. After centrifuging at 4 °C and 10,000×*g* for 10 min, the neutral invertase (pH 6.0) activity in the supernatant was determined using the colorimetric method of DNS at 540 nm^32^. Glucose was used as standard. The invertase activity was represented by the generation rate of reducing sugar per unit protein (mg mg^−1^ protein h^−1^).

Isocitrate dehydrogenase (ICDH) was measured using the enzyme extraction prepared for N metabolic enzymes as described below. The reaction system (pH8.0) contained 3.5 mM MgCl_2_, 0.4 mM NADP^+^, 0.55 mM isocitrate, and 88 mM imidazole^34^. The absorbance at 340 nm was monitored for 120 s and the enzyme activity was presented by the reduction rate of NADP^+^ per unit protein (nmo mg^−1^ protein min^−1^).

Protein content in enzyme extraction was measured using the Bradford method^35^.

### Nitrogen, ammonium, soluble protein and free amino acids assays

To quantify total leaf N, about 50 mg pulverized dry leaf samples were digested by H_2_SO_4_–H_2_O_2_ at 260 °C. Because NO_3_^−^ concentration was very low in the digesting solution, it is assumed that almost all the N form in the digesting solution was NH_4_^+^. NH_4_^+^ was measured to represent total N using the indophenol blue colorimetric method at 625 nm^36^. (NH_4_)_2_SO_4_ was used as standard.

Free NH_4_^+^ in leaves was extracted by homogenizing samples with deionized water at 4 °C. The homogenate was centrifuged at 4 °C and 10,000×*g* for 10 min. NH_4_^+^ in the supernatant was measured spectrophotometrically at 625 nm^36^ and (NH_4_)_2_SO_4_ was used as the standard.

Soluble protein in the frozen leaf samples were extracted by homogenizing with sodium phosphate buffer (pH 6.8). After centrifuging at 4 °C and 10,000×*g* for 10 min, soluble protein in the supernatant was measured by the Bradford method at 595 nm^35^, and bovine serum albumin (BSA) was used as standard.

To obtain free amino acids in the samples, 100 mg frozen leaf powder was ground with 10% acetic acid, and then centrifuged at 4 °C and 10,000×*g* for 10 min. Total free amino acids in the supernatant was measured using the triadone colorimetric method at 580 nm^37^.

### Assays of nitrogen metabolic enzymes

Frozen samples (100 mg) were homogenized with 50 mM Tris–HCl buffer (pH 8.0, containing 2 mM Mg^2+^, 2 mM DTT, and 0.4 M Suc). The homogenates were centrifuged at 4 °C and 10,000×*g* for 10 min, and the supernatant was used for measurement of the activities of N metabolic enzymes. Glutamine synthase (GS) activity was measured spectrophotometrically at 540 nm according to Zhang et al.^38^ and presented indirectly by the absorbance at 540 nm (A_540_) per unit protein per hour. Glutamate synthase (GOGAT) activity was determined as in Singh and Srivastava^39^. The reaction mixture contained 10 mmol *α*-ketoglutarate, 1 mmol potassium chloride, 37.5 mmol Tris–HCl buffer (pH 7.6), 0.6 mmol NADH, 8 mmol l-glutamine and 0.3 mL enzyme. The absorbance at 340 nm was monitored for 120 s and the activity of GOGAT was estimated by the oxidation rate of NADH per unit protein (nmo mg^−1^ protein min^−1^). Glutamic–oxalacetic transaminase (GOT) and glutamic–pyruvic transaminase (GPT) activities were measured according to Wu et al.^40^ and presented as the production rate of pyruvate per unit protein (μmol mg^−1^ protein 30 min^−1^).

### Statistic analysis

Samples in the same pot were mixed as a replication, and all the data were means of four replications. One-way ANOVA was employed to analyze the effects of exogenous Suc. Multiple comparisons among Suc concentration treatments were performed using the Duncan’s new multiple range test. Differences were considered statistically significant when *P* < 0.05.

## Results

### Effects of exogenous Suc on plant growth, root activity and chlorophyll

**Figure 1:**
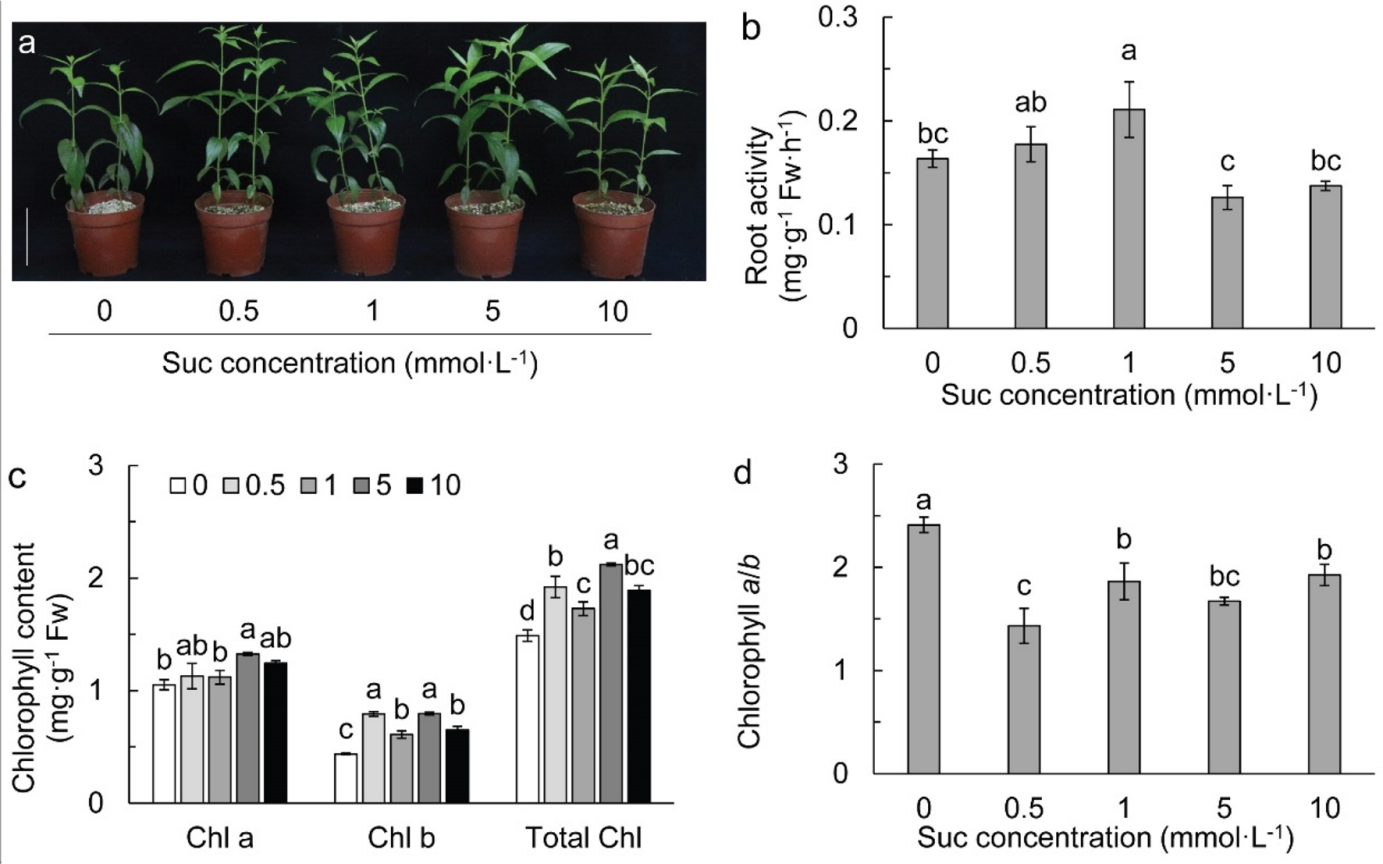
Plant phenotype (**a**), root activity (**b**), and chlorophyll (**c**,**d**) in different sucrose concentrations. Scale bar 10 cm in (**a**). Data refers to means ± SE (*n* = 4). Bars with the same letter are no statistically significantly difference at *P* < 0.05 by Duncan’s new multiple range method.

Figure 1a showed that supplying 0.5–5 mmol L^−1^ Suc in the nutritional growth media promoted plant growth observably, but 10 mmol L^−1^ Suc had no significant effect on plant growth. Root activity was significantly increased at 1 mmol L^−1^ Suc (Fig. 1b). Supply of 5 mmol L^−1^ Suc significantly increased chlorophyll *a* content; however, chlorophyll *b* and total chlorophyll were remarkably increased in all Suc treatments (Fig. 1c). As a result, the ratio of chlorophyll *a*/*b* was significantly reduced in Suc treatments (Fig. 1d).

### Effects of exogenous Suc on antioxidant indexes and anthocyanin accumulation

**Figure 2:**
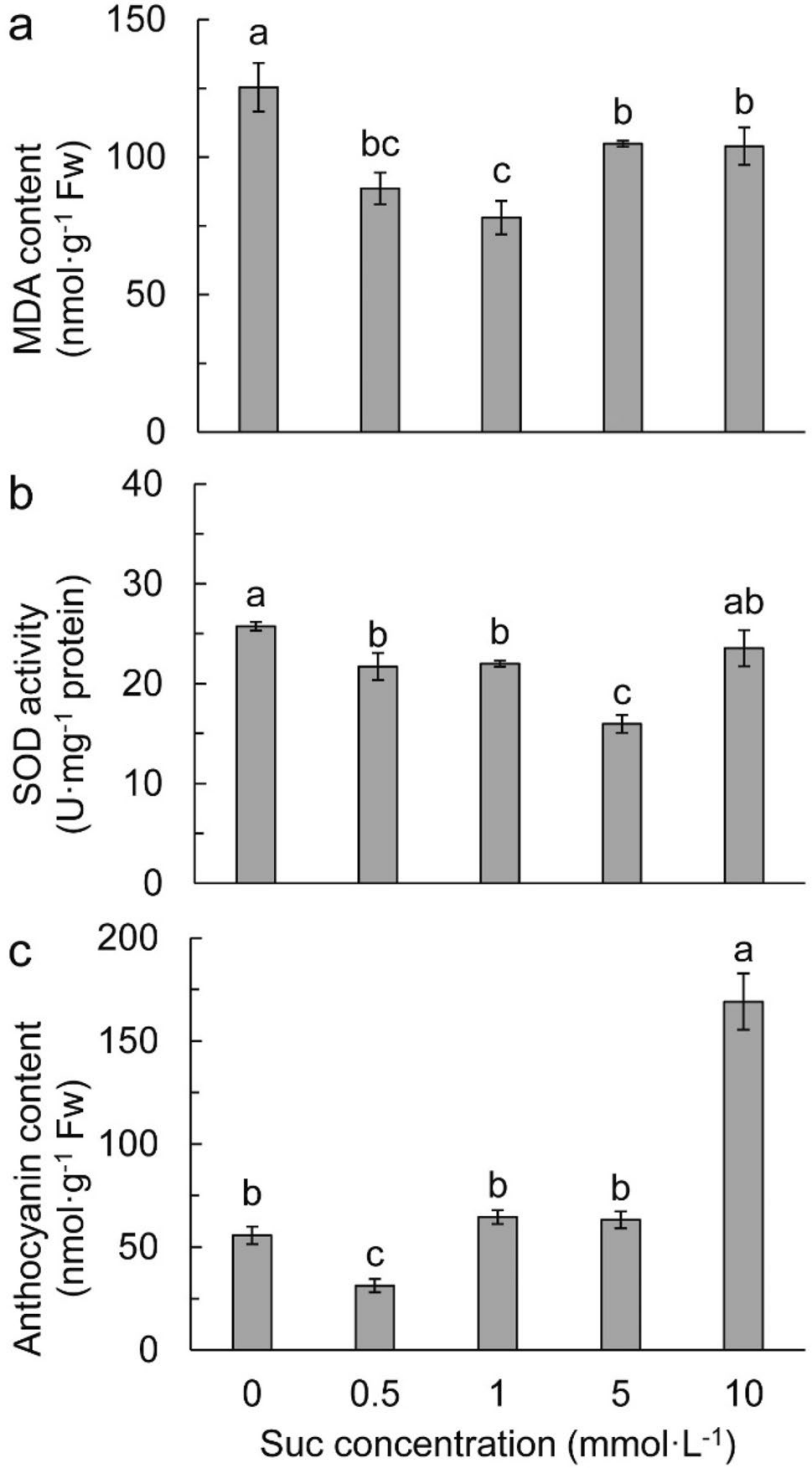
Leaf MDA content (**a**), SOD activity (**b**) and anthocyanin content (**c**) in different sucrose concentrations. Data refers to means ± SE (*n* = 4). Bars with the same letter are no statistically significantly difference at *P* < 0.05 by Duncan’s new multiple range method.

Leaf MDA content was remarkably lower in exogenous Suc treatments, but it was significantly increased in 5 and 10 mmol L^−1^ Suc in comparison to that in 1 mmol L^−1^ Suc (Fig. 2a). Supplying 0.5–5 mmol L^−1^ Suc significantly reduced SOD activity, but it was not different from the control in 10 mmol L^−1^ Suc (Fig. 2b). Compared with the control, 0.5 mmol L^−1^ Suc reduced anthocyanin accumulation remarkably; however, 10 mmol L^−1^ Suc induced a threefold increase in anthocyanin (Fig. 2c).

### Effects of exogenous Suc on nitrogen metabolism

**Figure 3:**
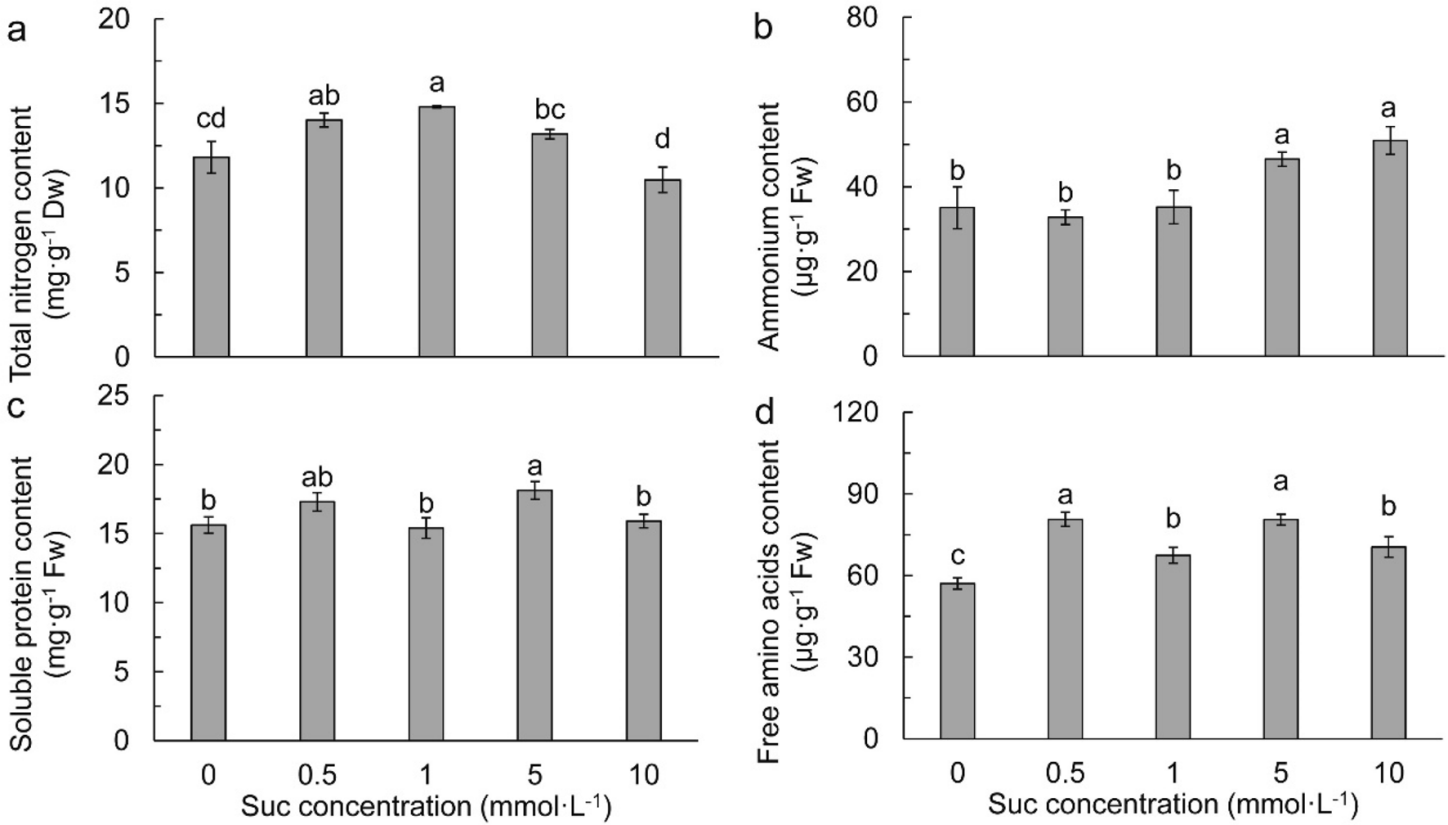
Leaf total nitrogen (**a**), ammonium (**b**), soluble protein (**c**), and free amino acids (**d**) in different sucrose concentrations. Data refers to means ± SE (*n* = 4). Bars with the same letter are no statistically significantly difference at *P* < 0.05 by Duncan’s new multiple range method.

As shown in Fig. 3a, low Suc concentrations (0.5 and 1 mmol L^−1^) increased leaf total N remarkably compared to the control. With a further increase of Suc concentration, total N content decreased gradually. It was significantly lower in 5 and 10 mmol L^−1^ Suc than in 1 mmol L^−1^ Suc but not different from the control. NH_4_^+^ content was not affected in 0.5 and 1 mmol L^−1^ Suc treatments, while it was remarkably increased in the treatments of 5 and 10 mmol L^−1^ Suc (Fig. 3b). Exogenous Suc of 5 mmol L^−1^ increased leaf soluble protein (Fig. 3c). Free amino acid was changed in line with soluble protein, but it was significantly higher in Suc treatments than in the control (Fig. 3d).

**Figure 4:**
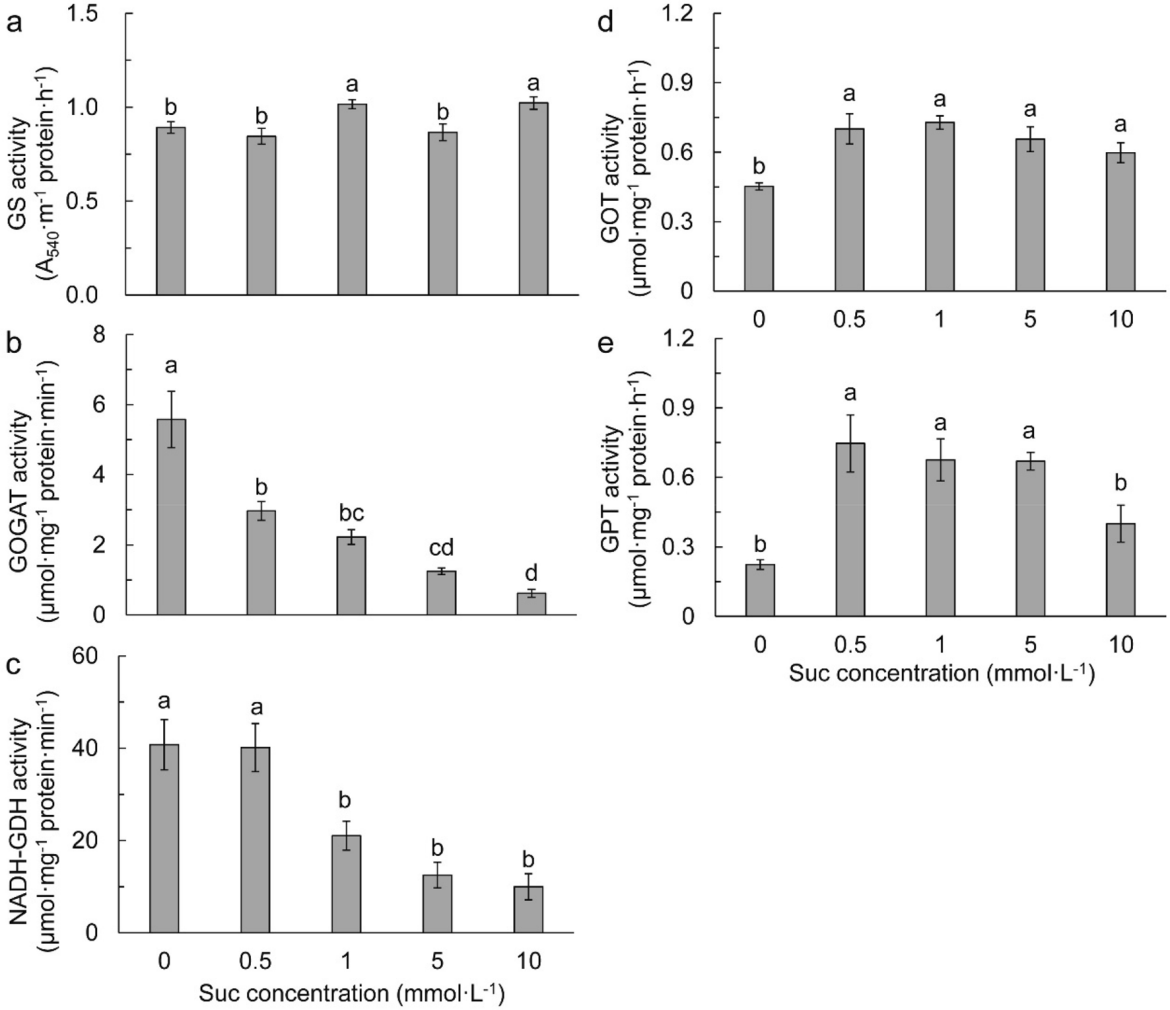
Glutamine synthase (GS, **a**), glutamate synthase (GOGAT, **b**), NADH-dependent glutamate dehydrogenase (NADH-GDH, **c**), glutamic–oxalacetic transaminase (GOT, **d**), and glutamic–pyruvic transaminase (GPT, **e**) activities in different sucrose concentrations. Data refers to means ± SE (*n* = 4). Bars with the same letter are no statistically significantly difference at *P* < 0.05 by Duncan’s new multiple range method.

GS activity was significantly increased by exogenous Suc at concentration of 1 and 10 mmol L^−1^ compared to the control (Fig. 4a). GOGAT activity was remarkably reduced with the increase of exogenous Suc concentration (Fig. 4b). NADH-GDH activity was significantly higher in the control and 0.5 mmol L^−1^ Suc, and decreased remarkably with the increase of Suc concentration (Fig. 4c). Exogenous Suc increased GOT activity (Fig. 4d), as well as GTP activity with the exception of 10 mmol L^−1^ Suc (Fig. 4e).

### Effects of exogenous Suc on sugar accumulation and metabolism

**Figure 5:**
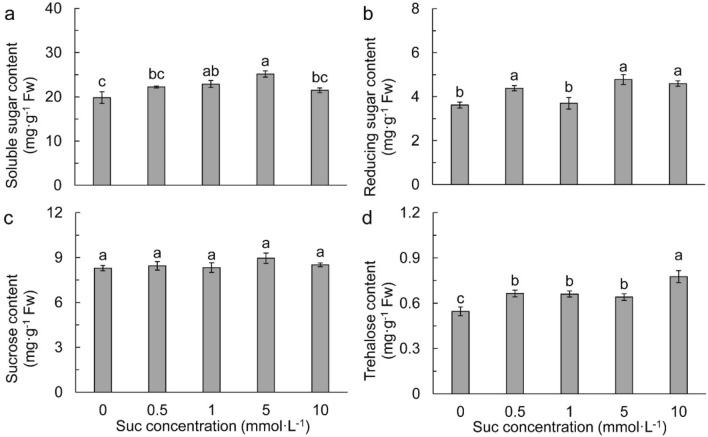
Total soluble sugar (**a**), reducing sugar (**b**), sucrose (**c**), and trehalose (**d**) in different sucrose concentrations. Data refers to means ± SE (*n* = 4). Bars with the same letter are no statistically significantly difference at *P* < 0.05 by Duncan’s new multiple range method.

With the increasing of exogenous Suc concentration, total soluble sugar increased and peaked at 5 mmol L^−1^ Suc, in which it was significantly higher than those in other treatments with the exception of 1 mmol L^−1^ Suc (Fig. 5a). Exogenous Suc increased reducing sugar in leaves with the exception of 1 mmol L^−1^ Suc (Fig. 5b). Nevertheless, endogenous Suc was not different among treatments (Fig. 5c). Trehalose was remarkably increased by Suc, with the highest in 10 mmol L^−1^ Suc (Fig. 5d).

**Figure 6:**
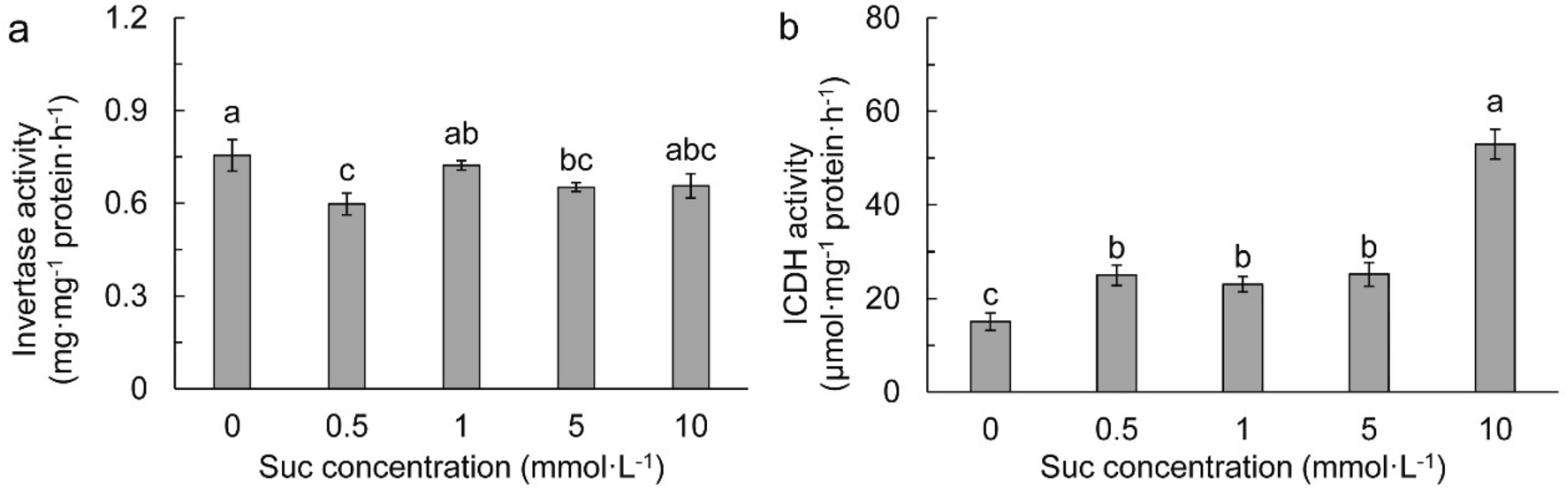
Invertase (IV, **a**) and isocitrate dehydrogenase (ICDH, **b**) activities in different sucrose concentrations. Data refers to means ± SE (*n* = 4). Bars with the same letter are no statistically significantly difference at *P* < 0.05 by Duncan’s new multiple range method.

Invertase (IV) activity was generally reduced by exogenous Suc, with a significant reduction in 0.5 and 5 mmol L^−1^ Suc (Fig. 6a). Isocitrate dehydrogenase (ICDH) activity was remarkably increased after supplementation of Suc, especially the 10 mmol L^−1^ Suc, in which it was about onefold higher than those in other Suc treatments (Fig. 6b).

## Discussion

### Dual effects of exogenous Suc concentration on plant growth

It has been widely reported that exogenous Suc affects differentially on plant growth, chlorophyll, photosynthesis, yield and quality in different plant species^21,22,41^. In this study, exogenous Suc concentration showed dual effects on the growth of Chuanxinlian plants, that is, it was promoted in 0.5–5 mmol L^−1^ Suc but retarded in 10 mmol L^−1^ Suc over the control. The results suggested that Suc with appropriate concentration is an important stimulant for the growth of Chuanxinlian regardless of its function as a nutrient for the plant or a stimulus for rhizospheric microbial reproduction. It has been reported that Suc-ameliorated plant growth was correlated with increased accumulation of soluble sugar, starch, N and amino acids^42,43^. Our findings were consistent with those studies that 0.5–5 mmol L^−1^ Suc induced improvement of plant growth was associated with increased accumulation of amino acids, soluble sugar and total N.

Exogenous application of Suc alters the photosynthetic apparatus, the activities of photosynthetic enzymes and the expression of relative genes^44^. Our results showed that chlorophyll *b* was significantly increased by exogenous Suc, suggesting exogenous application of Suc could startover the genes in chlorophyll *b* synthesis. The increase of chlorophyll *b* could expand the range of light intensity absorbed by plants to improve photosynthesis^45^. However, plant growth in 10 mmol L^−1^ was relatively inhibited (Fig. 1a). It could be due to the high anthocyanin concentration in leaves, because foliar anthocyanins shade the photosynthetic apparatus from light capture^46^ and therefore, reduce photosynthesis. Chlorophyll is an important organic N sink in plants^47^. The increase in chlorophyll *b* in the current study caused by exogenous Suc could be resulted from the changes in N metabolism.

Plant root is a heterotrophic organ which requires sugars derived from aboveground parts to provide energy for its physiological metabolism^48^. Increasing C supply for roots could promote N uptake and assimilation^49^. In this study, relative low Suc concentrations (e.g., 0.5 and 1 mmol L^−1^) increased root activity but high Suc concentration (10 mmol L^−1^) inhibited it, which was consistent with the difference in total leaf N content. It is suggested that Suc concentration-mediated plant growth was correlated with root metabolism promoting N uptake. The results also indicated that sugar supply is a key limiting factor for N uptake and assimilation in Chuanxinlian plants. Improving the photosynthetic capacity and concurrently promoting photosynthate transport from shoot to root are feasible measures to promote N utilization in Chuanxinlian plants.

### Exogenous Suc regulated balance of C and N metabolism

NH_4_^+^ is at the center of N metabolism flow in plant leaves^50^. High concentration of NH_4_^+^ in plant tissues is deleterious for most terrestrial higher plants^51,52^. It generally results in growth inhibition, disturbance of oxidative balance^53^, and even premature senescence^54^. Our results showed that NH_4_^+^ concentration in leaves was remarkably increased by 5 and 10 mmol L^−1^ Suc relative to other treatments, suggesting that high accumulation of NH_4_^+^ was responsible for high Suc concentration induced growth retardation of Chuanxinlian plants. NH_4_^+^ accumulation in plants could be resulted from several reasons such as protein degradation^55^, reduced generation of 2-oxoglutarate^56^, and reduced requirement for NH_4_^+^ due to accumulation of free amino acids^57^. In the conditions of 5 and 10 mmol L^−1^ Suc in this study, soluble protein and free amino acids were comparable to those in the conditions of 0.5 and 1 mmol L^−1^ Suc, suggesting that high Suc concentration induced NH_4_^+^ accumulation due to neither protein degradation nor reduced NH_4_^+^ requirement.

Assimilation of NH_4_^+^ requires 2-oxoglutarate (2-OG) to provide C skeletons. ICDH catalyzed dehydrogenation of isocitrate in the Krebs cycle is an important source of 2-OG^58,59^, which connects C and N metabolisms. In the current study, supplying of Suc increased leaf reducing sugar and ICDH activity, suggesting an increased supply of C-skeleton for NH_4_^+^ assimilation. The increase of ICDH activity was consistent with that of total N at low Suc concentration (0.5–5 mmol L^−1^). However, 10 mmol L^−1^ Suc reduced leaf N accumulation although the ICDH activity was greatly higher than that in other Suc concentration treatments. It is probably that high Suc concentration caused uncoupling of C and N metabolisms and thereby plant growth inhibition in Chuanxinlian.

The balance between NH_4_^+^ production and assimilation is important for plants to adapt to environments^60^. There are two distinct pathways of NH_4_^+^ assimilation in plants, namely the GS-GOGAT cycle and NADH-GDH pathway^61,62^. In this study, the remarkably reduced GOGAT and NADH-GDH activities were responsible for the highly accumulation of NH_4_^+^ in the conditions of 5 and 10 mmol L^−1^ Suc, indicating that high concentration of Suc could have disturbed NH_4_^+^ assimilation enzymes. Amino acid metabolism affects NH_4_^+^ accumulation in plants^52,57^. The GOT and GPT enzymes catalyze transamination of glutamate to aspartate and alanine, respectively, for the synthesis of branched-chain amino acids^63^. The relative low GOT and GPT activities in 10 mmol L^−1^ Suc could act somewhat as feedback inhibitory regulation of NH_4_^+^ assimilation and finally, resulted in high NH_4_^+^ but low total N accumulation.

### High Suc concentration caused plant senescence

High carbohydrate availability plays an important signaling role in regulating plant growth and development and metabolism, including leaf senescence^64^. Anthocyanin is an important indicator of plant senescence resulted from sugar accumulation in plant tissues^65^. During plant senescence, SOD activity and membrane lipid peroxidation are also increased^66^. In this study, supplying of Suc increased the availability of sugars in Chuanxinlian plants, whereas 10 mmol L^−1^ Suc remarkably increased the anthocyanin concentration in leaves relative to other treatments; simultaneously, MDA content and SOD activity were maintained at relatively high levels. Those results were in agreement with the phenotype of plant growth inhibition and suggested that such high Suc concentration induced leaf senescence^64^.

Trehalose metabolic pathway plays an important role in sensing C status in plants and regulating plant development^67^. It has been reported that exogenous application of Suc increased content of trehalose 6-phosphate (T6P) in plants and resulted in anthocyanin accumulation^68^. Strong accumulation of T6P is required for leaf senescence under the condition of high C availability^64^. Trehalose accumulation induced plant growth inhibition was associated with the increase of its precursor T6P^69^. Our results showed that trehalose content in Chuanxinlian plant was increased by supplying of Suc, with the greatest increase in 10 mmol L^−1^ Suc (Fig. 5d). It is speculated that the dual effects of exogenous Suc concentration on Chuanxinlian plant growth was associated with trehalose metabolism, and that there was a threshold of trehalose concentration in regulation of Chuanxinlian plant growth.

## Conclusion

**Figure 7:**
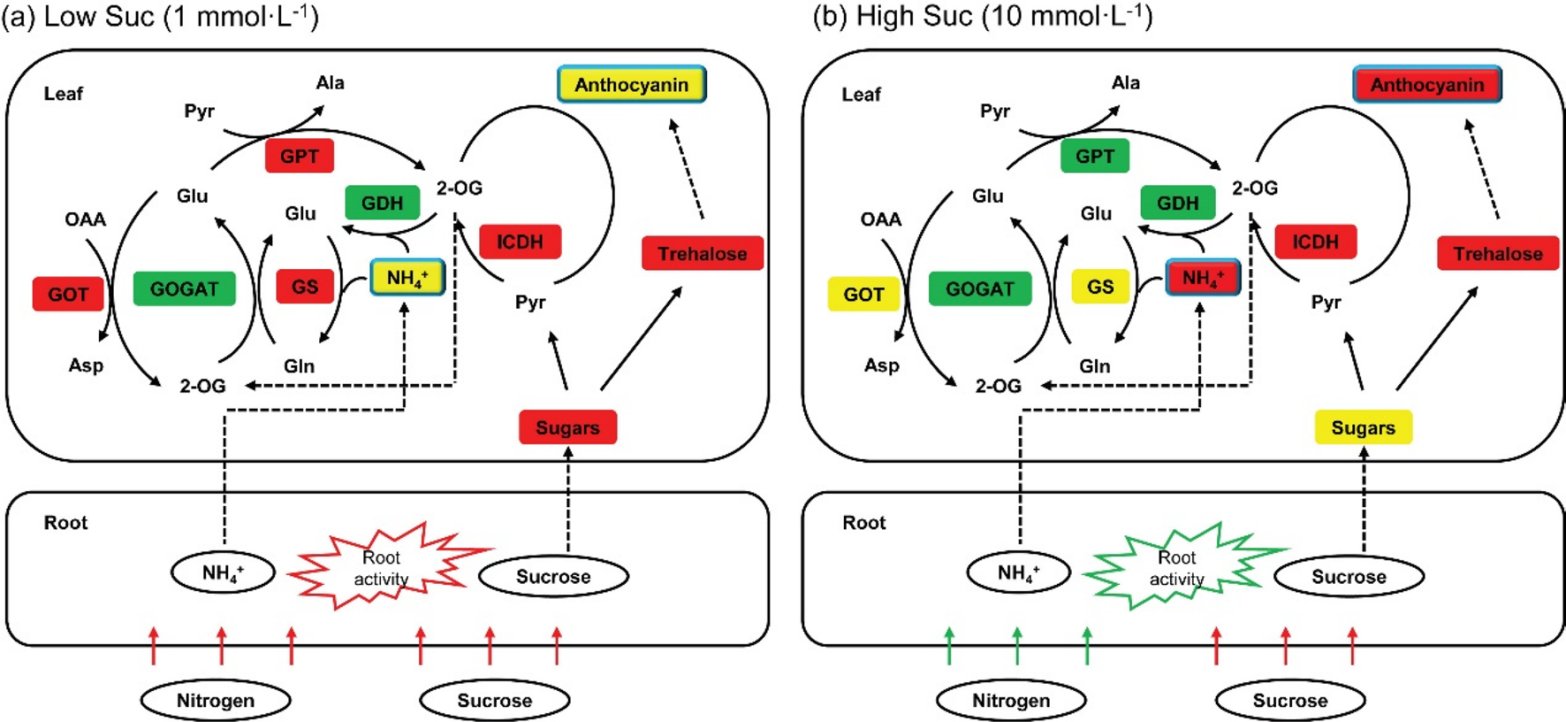
Simplified representation of the effect of exogenous Suc concentration on C and N metabolism in *A. paniculata*. (**a**) Effects of 1 mmol L^−1^ Suc on plant metabolites and enzyme activities in comparison to the control. (**b**) Effects of 10 mmol L^−1^ Suc on plant metabolites and enzyme activities in comparison to 1 mmol L^−1^ Suc. Red lines and boxes represent significant increase, green lines and boxes represent significant decrease, and yellow boxes represent unchanged. NH_4_^+^ and anthocyanin were highlighted by blue wireframe, because they are key products of N and C metabolism, respectively, whose changes were closely related to the differential responses of plants to exogenous Suc.

Our results revealed that exogenous application of Suc is an effective way to improve N utilization and growth of Chuanxinlian plants. However, it has dual effects on Chuanxinlian plant growth and N metabolism depending upon Suc concentration. An exogenous Suc concentration of less than 5 mmol L^−1^ could be appropriate to facilitate Chuanxinlian plant growth. In these conditions, exogenous Suc provided plants ample energy and C skeletons for N uptake and assimilation, resulting in increment of protein synthesis and growth amelioration. In contrast, 10 mmol L^−1^ Suc stimulated highly accumulation of NH_4_^+^ and anthocyanin due to uncoupling of C and N metabolism, and consequently inhibited both plant growth and N accumulation. Figure 7 exhibited the summarized schematic model of different Suc concentrations (i.e., 1 mmol L^−1^ and 10 mmol L^−1^) affecting C and N metabolism in Chuanxinlian plant.

## Abbreviations

2-OG: 2-Oxoglutarate
BSA: Bovine serum albumin
C: Carbon
DNS: 3,5-Dinitrosalicylic acid
Fe: Iron
GDH: Glutamate dehydrogenase
GOGAT: Glutamate synthase
GOT: Glutamic–oxalacetic transaminase
GPT: Glutamic–pyruvic transaminase
GS: Glutamine synthase
ICDH: Isocitrate dehydrogenase
IV: Invertase
MDA: Malondialdehyde
N: Nitrogen
PAR: Photosynthetic active radiation
SOD: Superoxide dismutase
Suc: Sucrose
T6P: Trehalose 6-phosphate
P: Phosphorus
